# Functional Interaction between Acyl-CoA Synthetase 4, Lipooxygenases and Cyclooxygenase-2 in the Aggressive Phenotype of Breast Cancer Cells

**DOI:** 10.1371/journal.pone.0015540

**Published:** 2010-11-11

**Authors:** Paula M. Maloberti, Alejandra B. Duarte, Ulises D. Orlando, María E. Pasqualini, Ángela R. Solano, Carlos López-Otín, Ernesto J. Podestá

**Affiliations:** 1 Instituto de Investigaciones Moleculares de Enfermedades Hormonales Neurodegenerativas y Oncológicas (IIMHNO), Department of Human Biochemistry, School of Medicine, University of Buenos Aires, Buenos Aires, Argentina; 2 Instituto de Biología Celular, School of Medicine, Córdoba National University, Córdoba, Argentina; 3 Instituto Universitario de Oncología, Department of Biochemistry and Molecular Biology, Oviedo University, Oviedo, España; University of Medicine and Dentistry of New Jersey, United States of America

## Abstract

The acyl-CoA synthetase 4 (ACSL4) is increased in breast cancer, colon and hepatocellular carcinoma. ACSL4 mainly esterifies arachidonic acid (AA) into arachidonoyl-CoA, reducing free AA intracellular levels, which is in contradiction with the need for AA metabolites in tumorigenesis. Therefore, the causal role of ACSL4 is still not established. This study was undertaken to determine the role of ACSL4 in AA metabolic pathway in breast cancer cells. The first novel finding is that ACSL4 regulates the expression of cyclooxygenase-2 (COX-2) and the production of prostaglandin in MDA-MB-231 cells. We also found that ACSL4 is significantly up-regulated in the highly aggressive MDA-MB-231 breast cancer cells. In terms of its overexpression and inhibition, ACSL4 plays a causal role in the control of the aggressive phenotype. These results were confirmed by the increase in the aggressive behaviour of MCF-7 cells stably transfected with a Tet-off ACSL4 vector. Concomitantly, another significant finding was that intramitochondrial AA levels are significantly higher in the aggressive cells. Thus, the esterification of AA by ACSL4 compartmentalizes the release of AA in mitochondria, a mechanism that serves to drive the specific lipooxygenase metabolization of the fatty acid. To our knowledge, this is the first report that ACSL4 expression controls both lipooxygenase and cyclooxygenase metabolism of AA. Thus, this functional interaction represents an integrated system that regulates the proliferating and metastatic potential of cancer cells. Therefore, the development of combinatory therapies that profit from the ACSL4, lipooxygenase and COX-2 synergistic action may allow for lower medication doses and avoidance of side effects.

## Introduction

The acyl-CoA synthetase, ACSL4 or FACL4, belongs to a five-member family of enzymes that esterify mainly arachidonic acid (AA) into acyl-CoA [1], [2]. A striking feature of ACSL4 is its abundance in steroidogenic tissues [1]. In contrast, ACSL4 is poorly expressed in other adult tissues, including breast, liver and the gastrointestinal tract in general [3], [4], [5]. Abnormal expression of ACSL4 in non-steroidogenic tissues has been involved in tumorigenesis [3], [4], [6]. In fact, ACSL4 overexpression has been reported in colon adenocarcinoma, hepatocellular carcinoma and breast cancer [3], [4], [5]. In human breast cancer, ACSL4 is differentially expressed as a function of estrogen receptor alpha status [5].

The release of AA has been indicated as an important signal leading to cellular proliferation. AA is, in turn, converted to different biologically active eicosanoid metabolites by three main enzymatic activities: lipooxygenase (LOX), cyclooxygenase (COX) and epooxygenase-cytochrome P450. LOX and COX are known to play a critical role in cancer progression i.e. growth and metastasis [7], [8], [9], [10].

Differences in abundance and activity of AA-converting enzymes may result in variations in the cellular content of eicosanoids. Therefore, and in view of the potential effects exerted by AA and derived eicosanoids, the enzymatic release of AA, its intracellular distribution and concentration are all under rigorous control within cells. Classically, activation of cytosolic phospholipase A2 has been considered as the rate-limiting step in the generation of AA. However, an alternative pathway that releases AA in specific compartments of the cell, e.g. mitochondria, has been described in steroidogenic tissues [11], [12], [13], [14]. This pathway, in which the rate-limiting enzyme is ACSL4, provides arachidonoyl-CoA to a mitochondrial acyl-CoA thioesterase (ACOT2) that releases AA in mitochondria and directs this fatty acid to the LOX enzyme for its subsequent conversion to lipooxygenase metabolites [15], [16]. In this pathway, the Translocator Protein (TSPO) [17], which resides in the outer mitochondrial membrane where it associates with the acyl-CoA binding protein DBI (diazepam binding inhibitor), is a crucial partner in the regulation of AA levels within the mitochondrion, from where it is exported for further conversion to eicosanoid products [16], [17].

As for ACSL4, altered TSPO expression has also been involved in several pathological conditions including breast, colon and liver cancer [18], [19]. Moreover, TSPO expression levels show a strong correlation with the development of the aggressive phenotype of different breast cancer cell lines [18], [20].

Increased ACSL4 expression, both at mRNA and protein levels [3], in colon adenocarcinoma cells has been associated with inhibition of apoptosis and increase in cell proliferation when compared to adjacent normal tissue. Based on those results, it has been suggested a role for ACSL4 in reducing free AA levels within cells and its association with apoptosis [3], [6], [21]. If this is the case, the reduction of free AA levels will impair the production of lipooxygenase and cyclooxygenase metabolites of AA that are known to potentiate tumor aggressiveness in colon adenocarcinoma [3], hepatocellular carcinoma [4] and breast cancer [5]. In addition, increased expression of cyclooxygenase-2 (COX-2) and of 5-lipooxygenase (5-LOX) has been reported in aggressive metastatic breast cancer cells [22], [23]. Thus, the role of ACSL4 in tumorigenesis enhancing the proliferation or invasive potential of the cells is not clear yet. Therefore, it is important to determine the role and molecular mechanism that govern the relationship between ACSL4 expression and the metabolic pathway of AA in cancer cells. To address this question, we used a breast cancer cell model to study the relationship between ACLS4 expression and intramitochondrial AA and its role in the generation of lipooxygenase and cyclooxygenase metabolites and the development of an aggressive cell phenotype.

We found that ACSL4 is significantly up-regulated in highly aggressive breast cancer cell lines, manifested as higher mRNA and protein abundance, as compared to less aggressive phenotypes in breast cancer cells. Functionally, we found that ACSL4 is part of the mechanism responsible for the increase in breast cancer cell proliferation, invasion and migration. We also found that the levels of LOX and COX products of AA are regulated by ACSL4 and that COX-2 expression is controlled by ACSL4 and lipoxygenated metabolites. In this mechanism, ACSL4, LOX and COX-2 interact functionally and represent an integrated system that regulates the proliferation and metastatic potential of cancer cells. This system seems to act in a concerted manner to promote breast cancer cell proliferation, invasion and migration.

## Results

### Expression and functional role of ACSL4 in breast cancer cell lines

**Figure 1:**
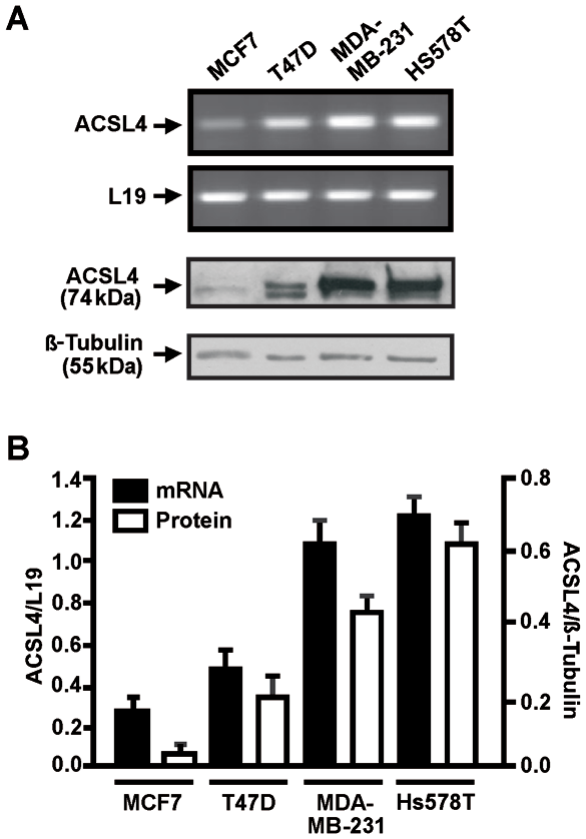
ACSL4 expression in human breast cancer cell lines. *A*: RT-PCR and Western blot analysis for ACSL4 mRNA and protein expression respectively, were performed as previously described [47]. Using anti-ACSL4 and anti-β-tubulin, specific protein bands were detected in immunoblots by enhanced chemiluminescence. *B*: The integrated optical density of PCR products was quantified and normalized with the corresponding L19 mRNA bands (closed bars). The integrated optical density of ACSL4 protein levels was quantified and normalized with the corresponding β-tubulin signal (open bars). Data represent the means ± SD of three independent experiments.

**Figure 2:**
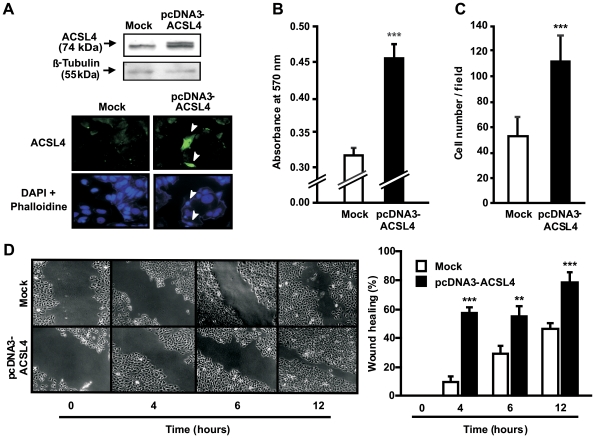
Effect of ACSL4 overexpression on cell proliferation, migration and invasion in MCF-7 cells. MCF-7 cells were transiently transfected with the plasmid containing ACSL4 cDNA (pcDNA3-ACSL4) or empty plasmid (mock). *A*: Forty-eight hours after transfection, ACSL4 protein levels were evaluated by Western blots and immunocytochemistry. The arrows indicate the effect of the transfection in single cells. *B*: Seventy-two hours after transfection, cell proliferation was measured by the MTT assay, as previously described [55]. *C*: Forty-eight hours after transfection, cellular invasion was measured by the matrigel assay, as previously described [57]. *D*: Forty-eight hours after transfection, cellular migration was measured by the wound healing assay, as previously described [56]. D left: contrast micrographs of cells. D right: At the specified time points, the distance between the wound edges was measured using Image-Pro Plus software. Data represent the means ± SD of three independent experiments. ** *P*<0.01 and *** *P*<0.001 vs. mock-transfected cells.

A breast cancer cell model comprising different cell lines with varying cell proliferation, invasive and metastatic behavior was used to investigate the possible effect of the differential expression of ACSL4 on the aggression phenotype. RT-PCR and immunoblot analysis (Fig. 1) showed that ACSL4 is expressed in all cell lines although at different levels, and that increased expression of the enzyme associates with the reported aggressive phenotype of each cell line. ACSL4 is usually detected as a duplet in Western blot experiments [24], [25], [26]. This is true for both wild type and transfected ACSL4 as observed in Figures 1 and 2. The gene encoding ACLS4 shows alternative splicing [24], [26] and the protein is phosphorylated (personal communication), and possibly acetylated. A member of the long chain acyl-CoA synthetase family, ACSL1, is acetylated at both the N-terminal and at a lysine residue and tyrosine residues are found to be phosphorylated [27]. It was also suggested that the different mobility is due to proteolisis or post-translational modifications such as N-glycosilation [24]. Both alternative splicing and post translational modification mechanisms can explain the detection of a duplet by Western blots.

We next analyzed the role of ACSL4 in cell growth, invasion and migration, in MCF-7 cells overexpressing ACSL4. As expected, transient transfection of MCF-7 cells with the cDNA coding for ACSL4 resulted in three-fold protein levels as determined by Western blot (Fig. 2A). First, we investigated the effect of ACSL4 overexpression on the proliferation rate of MCF-7 cells. ACSL4 increased the proliferation rate of MCF-7 cells, as compared to mock-transfected cells (Fig. 2B). ACSL4 overexpression resulted also in a significant increase in the invasion MCF-7 cells as compared to mock-transfected cells (Fig. 2C).

Overexpression of ACSL4 induced a more rapid cell migration towards the injury area in a wound healing assay. As observed in Figure 2D, differences in the wound area between ACSL4-transfected and mock-transfected cells were evident as early as 4 h after injury. In addition, ACSL4-overexpression induced a nearly complete closure of the wound after 12 h, whereas mock-transfected cells failed to cover the injury area in that time (Fig. 2D).

**Figure 3:**
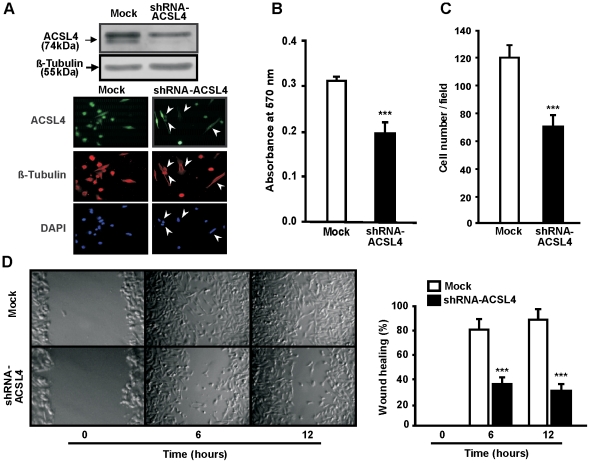
Effect of ACSL4 knock-down expression on cell proliferation, migration and invasion in MDA-MB-231 cells. MDA-MB-231 cells were transiently transfected with the plasmid containing shRNA-ACSL4 (shRNA-ACSL4) or an empty plasmid (mock). Forty-eight hours post-transfection, ACSL4 protein levels (*A*), proliferation (*B*), invasion (*C*) and migration (*D*) were evaluated as described in Fig. 2. Data represent the means ± SD of three independent experiments. *** *P*<0.001 vs. mock-transfected.

We further investigated the role of ACSL4 using small hairpin RNA (shRNA) to disrupt the expression of endogenous ACSL4 in the more aggressive cell line MD-MB-231. The shRNA targeting ACSL4 knocked down the expression of the protein approximately 40% (Fig. 3A), which in turn resulted in inhibition of cell proliferation (Fig. 3B), invasion (Fig. 3C) and migration (Fig. 3D) when compared to mock-transfected cells.

### Relationship between intramitochondrial AA, lipooxygenase product levels and aggressiveness of breast cancer cells

ACSL4 provides arachidonoyl-CoA to the acyl-CoA thioesterase, ACOT2, which releases AA in the mitochondrion and directs this fatty acid to the LOX enzyme for its subsequent conversion to lipooxygenase metabolites [11], [12]. To investigate whether a similar mechanism operates in breast cancer cells, we studied ACOT2 expression and intramitochondrial AA and lipooxygenase metabolites levels in the MDA-MB-231 and MCF-7 cell lines.

**Figure 4:**
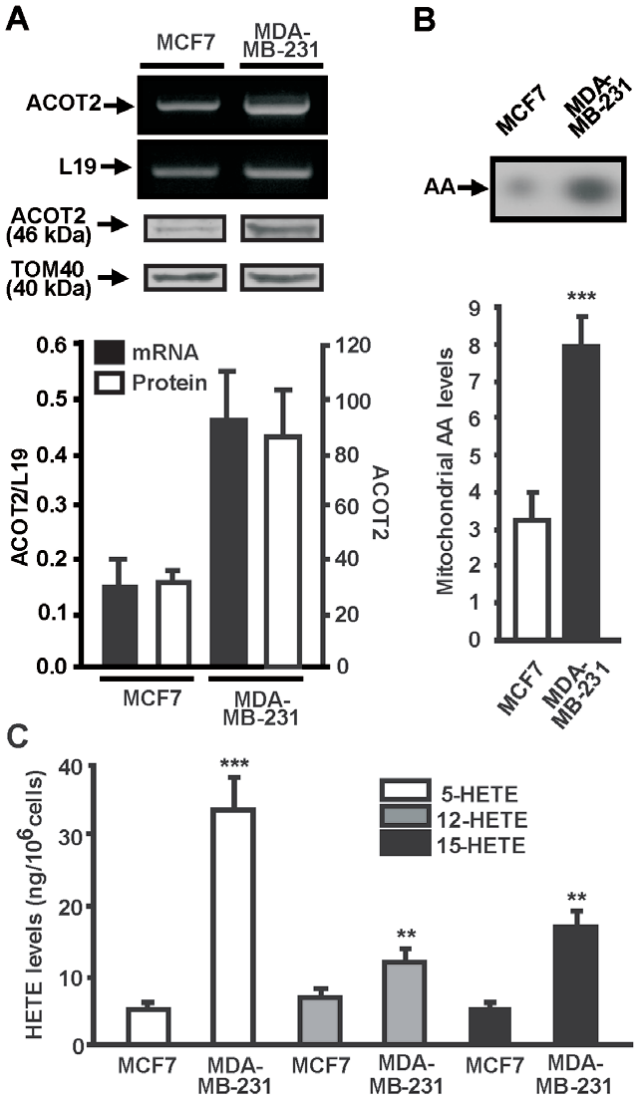
ACOT2, intramitochondrial AA, and of 5-, 12- and 15-HETE levels in breast cancer cells. *A*: Total RNA was extracted from MCF-7 and MDA-MB-231 cells and subjected to RT-PCR analysis for ACOT2 mRNA. Mitochondrial proteins were extracted from MCF-7 and MDA-MB-231 cells and evaluated by Western blot using an anti-ACOT2 antibody [48]. *B*: Representative autoradiography showing [1-^14^C] AA spots from mitochondrial fraction. [1-^14^C]-AA (New England Nuclear, Boston, MA, USA; specific activity 53.0 mCi.mmol-1) was added to the cultures in a concentration of 1 µCi.mmol-1per well (1×10^6^ cells) in serum-free DMEM containing 0.5% fatty acid-free bovine serum albumin. The mitochondria were isolated, sonicated and lipids were extracted and analyzed by thin layer chromatography (TLC) on silica gel plates, as previously described [12]. The autoradiographs were quantified by densitometry. Bars denote levels (in arbitrary units) of [1-^14^C] AA in mitochondria. Results are expressed as the means ± SD from three independent experiments. ***P<0.001. *C*: The levels of 5-, 12- and 15-(S)HETE accumulated for 48 h in the medium were determined by HPLC [50]. Bars denote levels of the lipooxygenase metabolites. Results are expressed as the means ± SD from three independent experiments. *** *P*<0.001, ** *P*<0.01 vs. MCF-7 cells.

ACOT2 expression was also evident in both cell lines (Fig. 4A). However, significant differences were observed in both mRNA and protein levels between the non-aggressive and aggressive cell lines when analyzed by RT-PCR or Western blot (Fig. 4A). Increased expression of ACOT2 associated with the aggressive cell phenotype was also observed.

In parallel with the expression of ACSL4 and ACOT2, intramitochondrial AA levels were significantly higher in MDA-MB-231 than in MCF-7 cells (Fig. 4B). A similar result was obtained when the levels of 5-, 12-, and 15-HETE (metabolites of 5-, 12-, and 15-LOX isoforms respectively) were measured (Fig. 4C). The levels of these three metabolites were significantly higher in MDA-MB-231 cells as compared to MCF-7 cells, with a 70-fold difference in favor of 5-HETE (Fig. 4C).

### Role of ACSL4 and ACOT2 in the production of lipooxygenase metabolites in breast cancer cells

**Figure 5:**
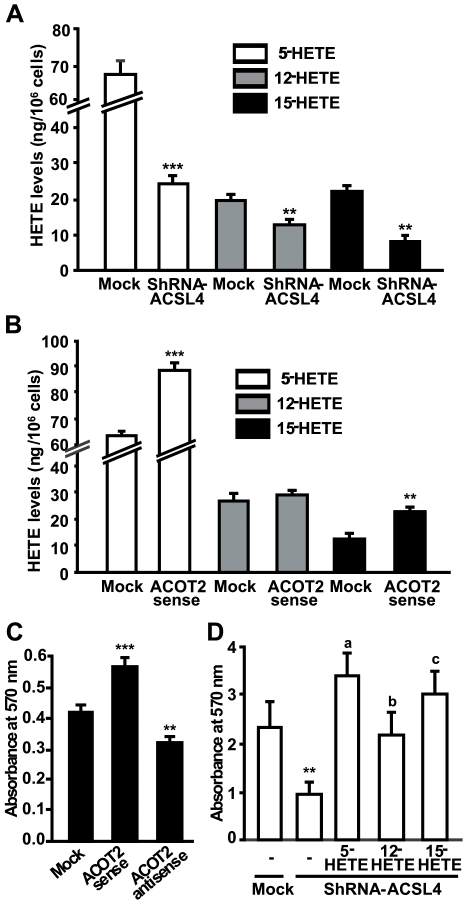
Effects of ACSL4 and ACOT2 expression on 5-, 12- and 15-HETE in MDA-MB-231 cells. *A*: MDA-MB-231 cells were transiently transfected with the empty plasmid (mock) or with shRNA-ACSL4. Forty-eight hours post-transfection, the levels of 5-, 12- and 15-HETE in the incubation media were analyzed by HPLC. *** *P*<0.001, ** *P*<0.01 vs. mock-transfected cells. *B*: MDA-MB-231 cells were transiently transfected with the empty pRCMVi plasmid (mock) or containing ACOT2 cDNA in the sense (ACOT2 sense) orientation. Seventy-two hours after transfection the lipooxygenase metabolites, 5-, 12- and 15-HETE were analyzed as described. Bars denote levels of the lipooxygenase metabolites. Results are expressed as the means ± SD from three independent experiments. *** *P*<0.001, ** *P*<0.01 vs. mock-transfected cells. *C*: MDA-MB-231 cells were transiently transfected with the plasmid containing ACOT2 cDNA in the sense (ACOT2 sense) or antisense (ACOT2 antisense) orientation Seventy-two hours after transfection, cell proliferation was measured by the MTT assay. Data represent the means ± SD of three independent experiments. *** *P*<0.001 and ** *P*<0.01 vs. mock-transfected. *D*: MDA-MB-231 cells were transfected as described in A. Forty-eight hours after transfection, the cell cultures were incubated with or without 10 µM of 5-, 12- and 15-HETE for 24 h. Cell proliferation was measured by the MTT assay. Data represent the means ± SD of three independent experiments. a, b, c *P*<0.001 vs. shRNA-ACSL4, ** *P*<0.01 vs. mock-transfected cells.

We then investigated the role of ACSL4 and ACOT2 in the production of lipooxygenase metabolites by disrupting the expression of endogenous ACSL4 and ACOT2 in MDA-MB-231 breast cancer cells. Inhibition of ACSL4 expression by shRNA decreased the levels of 5-, 12-, and 15-HETE (Fig. 5A) and resulted in inhibition on cell proliferation (Fig. 5D). The resultant reduction in cell proliferation was overcome by incubation of the cells with exogenous 5-, 12-, or 15-HETE (Fig. 5D). Changing the expression level of ACOT2 by sense and antisense treatment of MCF-7 cells had no effect on cell proliferation (data not shown). On the other hand, overexpression of ACOT2 in MDA-MB-231 cells increased the levels of lipooxygenase metabolites (Fig. 5B) and cell proliferation (Fig. 5C) whereas inhibition decreased the proliferation rate, as compared to mock-transfected cells (Fig. 5C).

**Figure 6:**
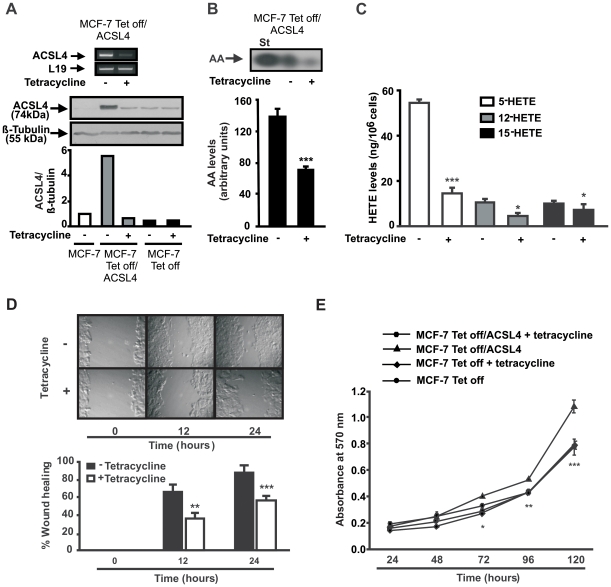
Effect of ACSL4 expression on cell migration and proliferation of MCF-7 Tet-off/ACSL4 cells. MCF-7, MCF-7 Tet-Off and MCF-7 Tet-off/ACSL4 cDNA cells were incubated with or without tetracycline. *A*: After 48 h of tetracycline treatment, total RNA was extracted and subjected to RT-PCR analysis for ACSL4 and L19 mRNA expression. Cellular proteins were obtained and subjected to SDS-PAGE and Western blot analysis. *B*: MCF-7 Tet-Off/ACSL4 cells were incubated with or without tetracycline. After 48 h, the cells were labeled with [1-^14^C] AA (1 µCi/ml) as described in fig. 4 and intramitochondrial [1-^14^C] AA levels were analyzed by TLC. Bars denote levels (in arbitrary units) of [1-^14^C] AA in mitochondria. Results are expressed as the means ± SD from three independent experiments. *** *P*<0.001 vs. without tetracycline. *C*: MCF-7 Tet-Off/ACSL4 cells were incubated with or without tetracycline. Forty-eight hours after transfection the lipooxygenase metabolites, 5-, 12- and 15-HETE were analyzed as described. Results are expressed as the means ± SD from three independent experiments. *** *P*<0.001, * P<0.05 vs. without tetracycline. *D*: Cell migration was measured by the wound healing assay at the indicated time points. Data represent the means ± SD of three independent experiments. *** *P*<0.001, ** *P*<0.01 vs. without tetracycline. *E*: Cell proliferation was measured by the MTT assay at the indicated time points. Data represent the means ± SD of three independent experiments. *** *P*<0.001, ** *P*<0.01 * *P*<0.05 vs. MCF-7 Tet-Off/ACSL4.

In order to confirm the results obtained by transient transfection, we stably transfected MCF-7 cells with ACSL4 using the tetracycline (Tet)-off system, as described in Materials and Methods. As expected, the MCF-7 Tet-off/ACSL4 cell line showed a significant increase in ACSL4 mRNA and protein, as compared with MCF-7 Tet-off control cells, and ACSL4 expression was completely repressed by the treatment of the cells with tetracycline, indicating that the system is fully functional in this cell line (Fig. 6A).

In this system, further functional studies were performed to confirm whether an increased ACSL4 expression may lead to increased cell proliferation and migration as described above. Indeed, MCF-7 Tet-off/ACSL4 proliferated at a much higher rate than control cells (Fig. 6E), an effect abrogated by the addition of tetracycline.

Tetracycline-treated confluent MCF-7 Tet-off/ACSL4 cells showed a significant delay in cell movement into the injury area as compared to controls when subjected to the wound healing assay (Fig. 6D). MCF-7 Tet-off/ACSL4 cells migrated into the wound area by 12 h, to such an extent that the wound edges were almost indistinguishable, whereas MCF-7 Tet-off/ACSL4 cells treated with tetracycline did not.

### Intramitochondrial AA content, formation of lipooxygenase products and expression of COX-2 in MCF-7 Tet-off/ACSL4 cells

As shown in Fig. 6B, intramitochondrial AA content in MCF-7 Tet-off/ACSL4 cells incubated in the presence of tetracycline was significantly different from that in those treated in the absence of tetracycline. The formation of 5-, 12-, and 15-LOX products was significantly increased in cells overexpressing ACSL4 than in cells treated with tetracycline (Fig. 6C).

**Figure 7:**
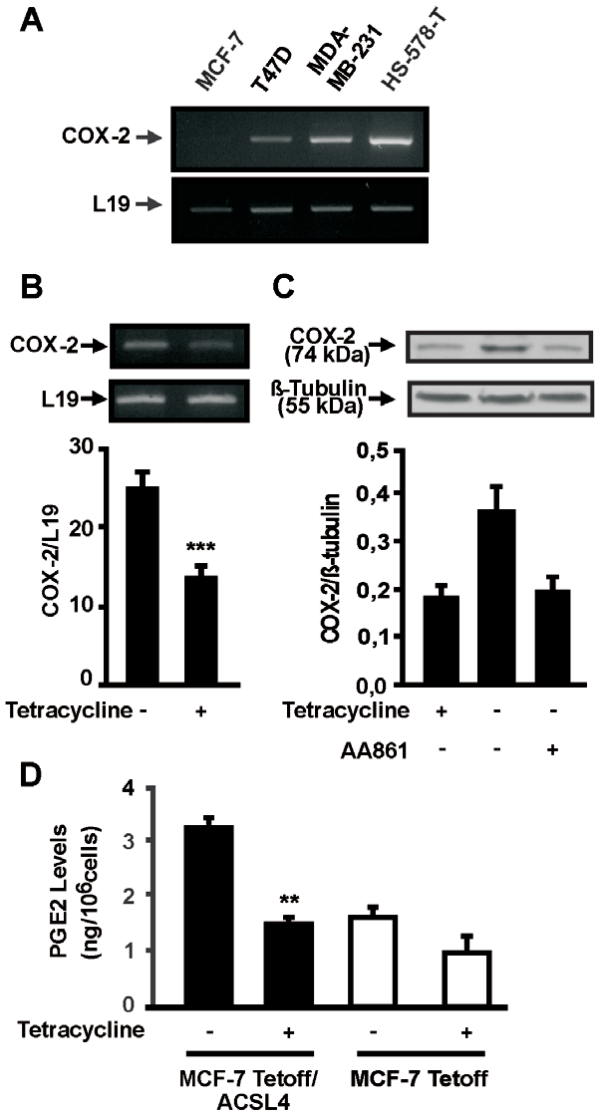
The effect of ACSL4 on COX-2 expression and prostaglandin biosynthesis. *A*: Total RNA was extracted from MCF-7, T47D, MDA-MB-231 and HS-578-T cells and subjected to RT-PCR analysis for COX-2 mRNA expression. *B*: MCF-7 Tet-off/ACSL4 cells were incubated with or without tetracycline. After 48 h of treatment, total RNA was extracted and subjected to RT-PCR analysis for COX-2 and L19 mRNA expression. Data represent the means ± SD of three experiments. *** *P*<0.001 vs. without tetracycline. *C*: MCF-7 Tet-off/ACSL4 cells were incubated in the presence or absence of the 5-LOX inhibitor, AA861 (20 µM). After 48 h of treatment, cellular proteins were obtained and subjected to SDS-PAGE and Western blot analysis using anti-COX-2 and anti-β-tubulin antibodies sequentially. Specific bands were detected by enhanced chemiluminescence. The integrated optical density of COX-2 protein levels were quantified for each band and normalized with the corresponding β-tubulin signal. *D*: MCF-7 Tet-off/ACSL4 or stably transfected with the empty vector were incubated with or without tetracycline. PGE_2_ accumulated for 48 h in the culture medium was analyzed by HPLC. Results are expressed as the means ± SD from three independent experiments. ** *P*<0.001 vs. without tetracycline.

Increased expression of COX-2 associates with an aggressive phenotype in breast cancer cell lines [28]. We therefore investigated COX-2 expression levels in the breast cancer cell model used for ACSL4 expression studies. RT-PCR showed expression of COX-2 in the different cell lines investigated (Fig. 7A). Cells with a more invasive potential, such as Hs-578-T and MDA-MB-231, displayed dramatically increased levels of COX-2 as compared to the non-aggressive lines MCF-7 and T47D. Transient transfection of MCF-7 cells with ACSL4 resulted in a significant increase in the expression of COX-2, thus indicating that ACSL4 expression associates with COX-2 expression (data not shown). Accordingly, COX-2 expression is upregulated in MCF-7 Tet-off/ACSL4 cells whereas tetracycline treatment of those cells reduced the expression of COX-2 (Fig. 7B).

To investigate whether the effect on COX-2 expression is mediated by the lipooxygenase products, we treated the cells with AA-861, a specific inhibitor of 5-LOX [29]. AA-861 blocked the effect in COX-2 levels caused by the overexpression of ACSL4 in the MCF-7 Tet-off/ACSL4 cell line (Fig. 7C). Nordihydroguayaretic acid, a more general LOX inhibitor, is also able to inhibit the effect of ACSL4 on COX-2 expression (data not shown). Finally, the increase in COX-2 expression in the MCF-7 Tet-off/ACSL4 cell line was accompanied by an increased production of PGE_2_ (Fig. 7D).

## Discussion

This study was undertaken to clarify the role of ACSL4 in tumorigenesis. We found that ACSL4 is a key enzyme in the mechanism of intramitochondrial AA generation and in the production of lipooxygenase and cyclooxygenase metabolites of AA in breast cancer cells. Our results, in terms of its overexpression and inhibition, demonstrate that ACSL4 plays a causal role in the control of breast cancer aggressiveness. Also, we report here for the first time, that ACSL4 controls the expression of COX-2 in those cells. Our results provide evidence of the involvement of ACSL4 in the mechanism responsible for the increase in proliferation, invasion and migration shown in breast cancer cells.

We found that ACSL4 mRNA and protein levels correlate with the aggressive phenotype in four different breast cancer cell lines. Consequently, transient transfection of the low aggressive phenotype and low-level expression of ACSL4 cell line, MCF-7, with the full length human ACSL4 cDNA resulted in increased aggressiveness of the cells manifested as augmented cell proliferation, invasion and migration. In line with these results, inhibition of ACSL4 expression in the highly aggressive and high-level expression of ACSL4 cell line, MDA-MB-231, by transfection of a plasmid containing ACSL4-shRNA resulted in decreased aggresiveness.

In a previous report [5] the knock down of ACSL4 in MDA-MB-231 cells did not change the growth rate of the cells. However, treatment of these cells with Triacsin C does result in a lower proliferation rate. The difference between the ACSL4 knock down experiment in this manuscript and the results reported by Monaco et. al. [5] may be explained by the protocol used. The protocol employed by Monaco includes a trypsination step after transfection and before measurement of cell proliferation rate. Trypsin treatment may change the proliferation rate as already described [30] [31], [32]. Thus, it can be argued that a longer culture time should be allowed after cell re-plating following transfection in order to evidence the effect of ACSL4 knock-down in cell proliferation rate. Moreover, in our experiments, we used shRNA instead of siRNA. shRNA constructs allow for sustained effects as compared to siRNAs [30], [31].

Our model of ACSL4 knock-down showed a very clear reduction in mRNA and protein levels that paralleled the reduction in cell growth. Moreover these results are further supported in the stable cells line where the inhibition of ACSL4 protein by tetracycline paralleled the inhibition of cell growth. Our results agree with those of Liang et al showing that ACSL4 is also associated with cell proliferation and that its knockdown blocks proliferation in hepatocellular carcinoma cells [33].

The inhibitory effect of Triacsin C on cell proliferation observed by Monaco et al could be the result of a synergistic effect of the inhibitor and of the siRNA. Triacsin C inhibits also ACSL1 and ACSL3 apart from ACSL4. However, no evidence is available showing that ACSL1 or ACSL3 activity increase cell proliferation. Moreover, in colon adenocarcinoma where ACSL4 expression is associated with an increased proliferation rate, ACSL1 expression was not changed or was even down-regulated [3]. As for ACSL3, it has been shown that the antiproliferative effect of Vitamin D in prostate cancer cells is mediated by ACSL3 overexpression, an effect that is blocked by Triacsin C [34]. Thus, an effect of Triacsin C on ACSL3 could only be proliferative. Our results using a shRNA and a Tet-off approach support the notion that ACSL4 up-regulation results in increased breast cancer cell proliferation rate. Altogether, the results obtained by Monaco et al using Triacsin C would also support the role of ACSL4 on cell proliferation.

The results obtained with these transient transfections were confirmed with the finding that MCF-7 Tet-off/ACSL4 cell line exhibited and increase in cell proliferation, invasion and migration. The finding that tetracycline abolished the difference in the aggressive phenotype of MCF-7 Tet-off/ACSL4 cells strongly indicates that the increase in cell proliferation, invasion and migration were a direct result of the increased ACSL4 expression. The time points for monitoring wound closure started when the wound was done. The difference observed in closure time between transiently-transfected (Fig. 2 and 3) and stably-transfected cells (Fig. 6) is explained by the fact that serum was present throughout the experiment in transient transfection while it was removed from the culture 24 h prior to wound infliction for stable transfectants (24 h serum starvation). Serum was not removed in transient transfection experiments in order to preserve cells from another stressor. On the other hand, stably-transformed cells are not subjected to transfection immediately before the wound experiment and it is, therefore, preferable to perform the assay with 24 h serum starvation prior to wound infliction to avoid cell growth during wound closure. Both approaches are accepted and used in the literature.

Another significant finding was that intramitochondrial AA levels were significantly increased in MDA-MB-231 cells as compared to those in MCF-7 cells and that increased AA levels correlated with higher expression levels of ACSL4. In addition we also found a 70-, 5-, and 3-fold increase in the levels of 5-, 15- and 12-HETE respectively in MDA-MB-231 as compared to MCF-7 cells. We have observed, concordant with what it is reported in the literature [28] a higher 5-LOX expression in MDA-MB-231 when compared to MCF-7 cells. However, overexpression of ACSL4 resulted in slight by not statistically significant increase in 5-LOX expression (data not shown). The involvement of ACSL4 in the regulation of intramitochondrial AA levels and lipooxygenase products were confirmed by inhibition of ACSL4 expression and the use of the MCF-7 Tet-off/ACSL4 transfection approach.

We also found that ACOT2 is significantly up-regulated in highly aggressive breast cancer cells. Interestingly, neither overexpression nor inhibition of ACOT2 expression in MCF-7, a cell that expresses low amounts of ACSL4, has an effect on cell proliferation. However, the phenotype was changed by both overexpression and knockdown of ACOT2 in MDA-MB-231 cells that express high levels of ACSL4. In addition overexpression of ACOT2 also increased the levels of the lipooxygenase metabolites. These results support the hypothesis that ACSL4 is the rate-limiting enzyme and that it works in combination with ACOT2 to generate intramitochondrial AA and the lipooxygenase metabolites as already reported for steroidogenic cells [11], [12], [13], [14].

In agreement with a previous work [28], we also found that COX-2 is expressed at much higher levels in MDA-MB-231 cells than in MCF-7 cells. Notably, the high abundance of COX-2 correlates with the higher expression levels of ACSL4. Stable transfection of MCF-7 cells with the full-length ACSL4 cDNA under the control of a tetracycline response element resulted in increased COX-2 constitutive expression. The finding that tetracycline abolished the expression of COX-2 strongly indicates that COX-2 expression is under the control of ACSL4 levels. In this work, we state that COX-2 expression in the more aggressive cells is controlled by ACSL4 through the generation of lipooxygenase metabolites since inhibition of lipooxygenase activity blunted ACSL4-dependent increase in COX-2 expression. This is an important finding that contributes to the understanding of the mechanism that controls COX-2 expression in breast cancer cells. The conclusion that ACSL4 may regulate COX-2 expression through the activation of lipoxygenase products is in line with previous observations showing that leukotriene B4, a product of 5-HETE, positively regulates the levels and stability of COX-2 mRNA and protein expression in synovial living cells [33]. Leukotrienes D4 (LTD4) is also known to stimulate COX-2 expression in intestinal and colon cancer cells [34].

Lipoxygenase inhibition can cause apoptosis. Thus, an apoptotic effect of AA861, other than its selective inhibition of lipoxygenase activity and causing a non-specific reduction in cell viability or survival rate, could be the cause of the attenuation of ACSL4 stimulation of COX-2. In our studies, we used a 20 µM concentration of AA861 during 48 h of incubation. In another study using MDA-MB-231 breast cancer cells, a 10 µM concentration of AA861 reduced cell proliferation rate in less than 10% only after 5 days of culture [22]. In the same study, this modest inhibition was only observed in two out of five different cell lines assayed [22]. Other cancer cell lines, such as HT29 human colon cancer cells, the effect of AA861 on survival and proliferation was only apparent with concentrations of AA861 above 30 µM [35]. Furthermore, a significant inhibition of cell growth of bladder cancer cells is reported with 20 µM AA861 again only after 5 days of culture [36]. It is therefore unlikely that the inhibition of ACSL4-stimulated COX-2 expression by AA861 reported here may be due to an activation of apoptosis or inhibition of cell growth or survival since the inhibition of COX-2 expression is evident at least 48 h earlier than the inhibition in cell proliferation described for different cancer cell lines. Also, since COX-2 inhibitors are known to induce apoptosis [37] a causal relationship between lipoxygenase and COX-2 inhibition with apoptosis seems plausible.

**Figure 8:**
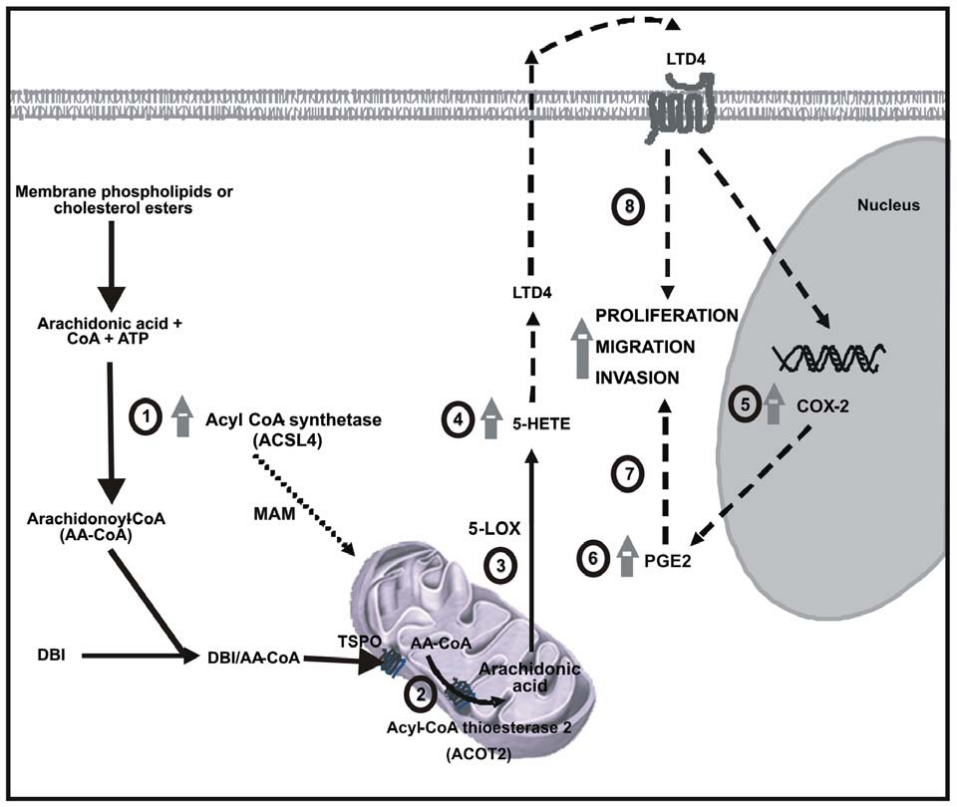
Schematic model of the signaling pathway involved in ACSL4-induced up-regulation of intramitochondrial AA, LOX products, COX-2 and PGE_2_ production in the development of an aggressive phenotype in breast cancer cells. Numbers (1-8) represent the schematic sequence of events that regulate cell proliferation, migration and invasion. 1, ACSL4 induction and action; 2, Acot2 induction and action; 3, 5-LOX action; 4, AA metabolism and function; 5, COX-2 induction and action; 6, PGE_2_ synthesis; 7, PGE_2_ action; 8, LOX metabolites action.

Previous investigations in colon adenocarcinoma or hepatocellular cells reported that ACSL4 overexpression or inhibition associated only with increased or decreased cell proliferation respectively [3], [4], [6], [21], [38]. The authors pointed to reduced free AA levels caused by ACSL4-mediated esterification results in reduced apoptosis and increased cell growth. We demonstrate here for the first time that the generation of prostaglandins is dependent on the presence of ACSL4. Thus, the esterification of AA by ACSL4 provides a way to compartmentalize the release of AA in the mitochondrion, a mechanism that serves to drive the specific biotransformation of the fatty acid instead of reducing the levels of free AA as previously suggested [3], [6], [21]. This, in turn, suggests that ACSL4 regulates the breast cancer cell phenotype through the production of lipooxygenase and cyclooxygenase metabolites. This may indicate that the regulation of proliferation, migration and invasion could be due to the functional interaction of ACSL4, LOX and COX2. An schematic representation of the proposed mechanism is shown in Figure 8.

Several LOX products have been shown to stimulate oncogenes and to produce mitogenic, antiapoptotic and chemotatic effects. In recent years the participation of 5-LOX in the regulation of cell proliferation and apoptosis has emerged [39]. In addition to normal cells, 5-LOX is expressed by a broad variety of cancer cells including breast cancer cells [22], [40], [41], [42]. Also, a much higher level of 12-LOX mRNA has been shown in tumorous cells as compared to normal cells in human breast cancer sections [43].

COX-2 has been suggested as a necessary component of the cellular and molecular mechanisms behind breast cancer cell motility and invasion [44], [45]. Consequently, it has been shown that infiltrating mammary carcinoma and intraductal carcinoma express significant levels of COX-2 whereas normal breast tissues do not [46]. COX-2 expression is strongly correlated with increased tumor microvascularity density and plays an important role in inhibiting apoptosis, stimulating angiogenesis and promoting tumor cell metastasis and invasion [45]. Since ACSL4 controls the expression of COX-2 through the induction of lipooxygenase metabolites of AA, as stated above, the functional interaction of COX-2 and LOX enzymes could have therapeutic implications. It has been suggested that COX-2 and 5-LOX may have redundant functions in cancer pathobiology. Romano and Claria [39] have raised the question of whether, if co-expressed, these arachidonic-acid-metabolizing enzymes represent an integrated system that regulates the proliferation and pro-angiogenic potential of cancer cells. We here provide evidence for such mechanism, in which ACSL4, LOX and COX-2 metabolites interact functionally. This interaction leads to the regulation of cell proliferation, migration and invasion with ACSL4 as the rate-limiting enzyme for intramitochondrial AA generation and export, for the conversion to lipooxygenase metabolites and the induction of COX-2.

Previous reports have demonstrated that the simultaneous activation of the ACSL4 and COX-2 pathways results in a synergistic anti-apoptotic effect in colon cancer [21]. Moreover, a combination of classical non-steroideal anti-inflammatory drugs aimed at inhibiting COX-2 and Triacsin C targeting ACSL4 has shown a synergistic pro-apoptotic effect in the colon cancer cell line HT29 [21]. In this study, we showed that ACSL4 acts in combination with ACOT2, LOX and COX-2 to generate an aggressive phenotype in breast cancer cells. Therefore, this combined action might lead to the development of therapies that profit from the ACSL4, LOX and COX-2 synergistic action and allow for lower medication doses and avoidance of side effects.

It can therefore be argued that ACSL4 overexpression might contribute to the development of an aggressive phenotype in breast cancer cells by regulating the production of lipooxygenase and cyclooxygenase metabolites. This might bring about a common mechanism, at least in tumors known to express high amounts of ACSL4, such as breast, colon and hepatocellular carcinoma, where its high expression is associated with tumorigenesis.

## Materials and Methods

Dulbecco's modified Eagle medium (DMEM) and penicillin-streptomycin solution trypsin-EDTA, G418 (Geneticin), Opti-MEM were from GIBCO, Invitrogen Corporation (Grand Island, NY, USA). Fetal Calf Serum was from PAA laboratories GmbH (Pasching, Austria). AA861 (2-(12-Hydroxydodeca-5,10-diynyl)-3,5,6-trimethyl-1,4-benzoquinone), 12(S)-hydroxyeicosatetraenoic acid (12(S)-HETE); 15(S)-hydroxyeicosatetraenoic acid (15(S)-HETE), 5-S-hydroperoxy eicosatetraenoic acid (5(S)-HETE), 7-[3-hydroxy-2-(3-hydroxyoct-1-enyl)- 5-oxo-cyclopentyl] hept-5-enoic acid (PGE_2_) were from Biomol (Plymouth Meeting, PA, USA). Puromycin, tetracycline and 3-(4,5-dimethyl-2-thiazolyl)-2,5-diphenyl-2H-tetrazolium bromide (MTT) were purchased from Sigma Chemical Co (St. Louis, MO, USA). Polyclonal rabbit anti-human ACSL4 and anti-human ACOT2 antibodies were generated in our laboratory [47], [48], whereas monoclonal mouse anti-β-tubulin was from Upstate Group Inc (Temecula, CA, USA). Polyclonal mouse anti-COX-2 antibody was from Cayman Chemical (Ann Arbor, MI, USA). Polyclonal rabitt anti-TOM40 (H-300): SC-11414 was from Santa Cruz Biothecnology, Inc. Horseradish peroxidase-conjugated goat-anti-rabbit and goat-anti-mouse secondary antibodies, polyvinylidene fluoride membrane was from Bio-Rad Laboratories (Hercules, CA, USA). Immun-Blot PVDF Membrane was from Bio-Rad Laboratories (Hercules, CA, USA). Enhanced chemiluminescence (ECL) was from GE Healthcare (Buckinghamshire, UK). Lipofectamine 2000 was from Invitrogen (Carlsbad, CA, USA). TriZol reagent was Molecular Research Center (Cincinnati, OH).

### Cell cultures

Human breast cancer cell lines MDA-MB-231, MCF-7, T47D, HS-578-T, were generously provided by Dr. Vasilios Papadoupoulus (Research Institute of the McGill University Health Centre, Montreal, Canadá) and obtained from the Lombardi Comprehensive Cancer Center (Georgetown University Medical Center, Washington D.C. USA). The cell lines were maintained in DMEM medium supplemented with 10% FBS plus 100 U/ml penicillin and 10 µg/ml streptomycin (complete DMEM).

### RNA extraction and semiquantitative RT-PCR

Total RNA was extracted using TriZol reagent (Molecular Research Center) following the manufacturer's instructions. Primers used for ACOT2 amplification (amplicon size 870 bp) were: sense primer, 5′-AGATCATTAGGGTTCCTGCTCG-3′ and the antisense primer, 5′-TTGATGCGATTTCTGTTGACG-3′. For ACSL4 amplification (amplicon size 115 bp), the sense primer, 5′-GAAGGTAAAAAGTTAACAGGCAAACAT-3′, and the antisense primer, 5′-TCAGAGTTTAAATCTCTTTCCCAGGTT-3′, were used. Primers used for amplification of the 406-bp segment of COX-2 were: sense: 5′-GCTCAGCCATACAGCAAATCC-3′, and antisense: 5′-GGGAGTCGGGCAATCATAAG-3′. The amplified L19 ribosomal protein product of each sample (amplicon size 500 bp) were used as housekeeping gene [49]. Specific primers for L19 were: sense: 5′-AGTATGCTCAGGCTTCAGAA-3′, and antisense: 5′-TTCCTTGGTCTTAGACCTGC-3′. The reaction conditions were one cycle of 94°C for 5 min, followed by 37 cycles for ACSL4 or 25 for L19 of 94°C for 30 sec, 60°C for 30 sec, and 72°C for 45 sec, 32 cycles for ACOT2 of 94°C for 30 sec, 60°C for 30 sec, and 72°C for 1 min. The number of cycles used for COX-2 was optimized independently for MDA-MB-231 (33 cycles) and MCF7 Tet-Off/ACSL4 (35 cycles) followed by 94°C for 30 sec, 60°C for 30 sec, and 72°C for 45 sec. The number of cycles used was optimized for each gene to fall within the linear range of PCR amplification. PCRs for ACOT2 amplification included 5% dimethylsulfoxide (DMSO). PCR products were resolved on 1.5% (wt/vol) agarose gels containing ethidium bromide. The gel images were digitally recorded and the amplicons levels were quantitated using the computer-assisted image analyzer Gel-Pro (IPS, North Reading, MA).

### Plasmid transfection

For transient transfection of MDA-MB-231 and MCF-7, cells were seeded the day before and grown up to 80% confluence. Transfection was performed with the pcDNA3.1(+) (Invitrogen) plasmid containing ACSL4 cDNA, the pSUPER.retro plasmid (OligoEngine) containing shRNA ACSL4 (5′ AAGATTATTCTGTGGATGA 3′) or the empty vectors as control in Opti-MEM medium and Lipofectamine 2000 reagent (Invitrogen). Transfection efficiency was estimated at approximately 40% as estimated by counting fluorescent cells transfected with the pRc/CMVi [11] plasmid containing the enhanced form of green fluorescent protein.

### Analysis of AA metabolites

12(S)-HETE; 15(S)-HETE, 5(S)-HETE and PGE_2_ were analyzed in the culture media of MDA-MB-231, MCF-7 and MCF-7 Tet-Off/ACSL4 cell lines by HPLC as previously described [50]. Briefly, 1×10^6^ cells were seeded in tissue culture dishes and incubated for 48 h in 2 ml of complete DMEM. The AA metabolites released to the culture media were extracted using Strata C18-T cartridges (Phenomenex, Torrance, CA, USA), eluted with 100% methanol. The solvent was evaporated and the residue was reconstituted in 0.1 ml of solution of methanol/water, 1∶2 v/v, and injected into a Hewlett-Packard 1100 HPLC connected to a UV detector. Quantification of AA metabolites was obtained by means of standard curves containing the same synthetic compounds (Biomol Co).

### Stable transfection of MCF-7 Tet-off cells with ACSL4 cDNA

The tetracycline-repressible MCF-7 cell line, designated MCF-7 Tet-off, was used for stably transfection of ACSL4 cDNA under control of the tetracycline-response element using the Tet-Off Gene Expression System (Clontech). After transfection, cells were maintained in complete DMEM supplemented with 0.3 µg/ml puromycin. After several weeks, colonies in which the expressed ACSL4 was regulated by tetracycline (2 µg/ml) were selected, cultured and subjected to different functional assays. MCF-7 Tet-off-induced repression of ACSL4 cells, designated MCF-7 Tet-off/ACSL4, were further maintained in complete DMEM.

### Western blot

Total or mitochondrial proteins (20 µg) were separated on SDS-PAGE and electro-transferred to poly(vinylidene difluoride) membranes (Bio-Rad Laboratories) as described previously [11]. Membranes were then incubated with 5% fat-free powdered milk in 500 mM NaCl, 20 mM Tris-HCl (pH 7.5), and 0.5% Tween 20 for 60 min at room temperature, with gentle shaking. The membranes were then rinsed twice in 500 mM NaCl, 20 mM Tris-HCl (pH 7.5), and 0.5% Tween 20 and incubated overnight with the appropriate dilutions of primary antibody at 4°C: 1∶500 rabbit polyclonal anti-ACOT2, 1∶1,000 rabbit polyclonal anti-ACSL4, 1∶5,000 mouse monoclonal anti-β-tubulin, and 1∶400 rabbit polyclonal anti-Cox-2. Bound antibodies were developed by incubation with secondary antibody 1∶5,000 goat anti-rabbit and 1∶5,000 goat anti-mouse horseradish peroxidase conjugated and detected by chemiluminescence. The inmunoblots were then quantitated using Gel Pro Analyzer.

### Isolation of mitochondria

Isolation of mitochondria was done as described [12]. Briefly, cell cultures were washed with PBS, scraped in 10 mM Tris-HCl (pH 7.4), 250 mM sucrose, 0.1 mM EDTA, 10 µM leupeptin, 1 µM pepstatin A, and 1 mM EGTA (buffer A), homogenized with a Pellet pestle motor homogenizer (Kimble Kontes, Vineland, NJ), and centrifuged at 600× g for 15 min. The supernatant obtained was centrifuged at 10,000× g for 15 min and rendered a mitochondrial pellet that was washed once with buffer A and resuspended in 10 mM Tris-HC (pH 7.4), 10 µM leupeptin, 1 µM pepstatin A, and 1 mM EGTA. Fractions were subjected to enzymatic analysis to assess their purity (according to [51], [52]). The purity of each fraction was at least 90%, value similar to previous publications [53].

### [1-^14^C] AA incorporation in breast cancer cells

Cells were labeled according to previously described methodology [54], with minor modifications [12]. [1-^14^C]AA (NEN Life Science Products, Boston, MA; specific activity 53 mCi/mmol) was added to the cultures (1 µCi/ml per well; 1 well  = 2×10^6^ cells) in serum-free culture media containing 0.5% fatty acid-free BSA. After 5 h of incubation at 37°C in a humidified atmosphere containing 5% CO_2,_ cells were washed with serum-free culture medium containing 0.5% fatty acid-free BSA. A steady state level is attained between 3 to 4 h, with an uptake of around 85%. The uptake reaches a plateau and it is sustained for 24 h. This uptake is similar in MCF-7 and MDA-MB-231 cells and is in accordance with previous observations in other cells. Mitochondrial pellets were obtained and resuspended as described above and were then sonicated. Protein concentration was measured and lipids were extracted from equal amounts of mitochondrial proteins (500 µg) from each treatment having previously added 500 ng of unlabeled AA.

Lipid extraction was performed twice with ethyl acetate (six volumes per one volume of mitochondrial fraction). The organic phase was then collected and dried under nitrogen at 25°C and analyzed by two successive thin-layer chromatographies on silica gel. Radioactive spots were developed using a Storm phosphorimager (Amersham Biosciences, Stockholm, Sweden) after 1 week of exposition, and the spot intensities were analyzed using ImageQuant

### Cell proliferation assay

Cell proliferation was measured by the MTT assay, as previously described [55]. Cells were plated at a density of 4000 cells/well in 96-well plates with 10% FBS-supplemented D-MEM medium and allowed to adhere overnight at 37°C in a humidified, 5% CO_2_ atmosphere. The medium was then changed to serum-free medium. After 24 h, the cells were switched to 10% FBS-supplemented D-MEM medium and incubated for the appropriate times as described in figure legends. Subsequently, MTT was added and incubated for 2∶30 h at 37°C. Next, the formed formazan crystals were dissolved with DMSO. The absorbance 570 nm was determined using a Multi-detection microplate reader, Synergy HT, Biotek (Winooski, Vermont, USA).

### Wound-healing assay

Cellular migration was measured by the wound healing assay, as previously described [56]. Cells (7×10^5^ cells per well) were seeded in six-well plates. Stable-transfectants were serum-starved for 24 h after which media was replaced (10% FBS medium) and the wound performed. Transiently-transfected cells were kept in complete (10% FBS) medium at all times. Wound infliction was considered as 0 time and wound closure monitored for up to 24 h wound closure. Cell monolayer was wounding with a plastic tip across the monolayer cells. Wound closures were photographed by a phase contrast microscopy (40X) in the time point 4, 6, 12 and 24 h after scraping. The width of the wound was determined with the program Image Pro-Plus.

### Invasion assay

Cellular invasion was measured by the matrigel assay, as previously described [57], with minor modifications. The *in vitro* invasion potential was evaluated using Matrigel-coated invasion chambers with an 8-µm pore size (BD Biosciences). Briefly, 5×10^4^ cells/ml were allowed to migrate for 22 h through the Matrigel-coated membranes using 5% fetal bovine serum (FBS) as chemoattractant. Cells that reached the lower surface of the membrane were stained and counted The number of invaded cells in six randomly selected microscopic fields per membrane was counted using an epifluorescence microscope Olimpus BX50 with a digital camera Cool/Snap Proof Color PM-c35.

### Statistics

Statistical analysis was performed by Student's t test or ANOVA followed by the Student-Newman-Kuels test.
